# Practical Observations on the Various Forms of Dyspepsia

**Published:** 1845-08

**Authors:** Robert Dick

**Affiliations:** Author of a Treatise on the “Derangements, Primary and Reflex, of the Organs of Digestion,” “A Treatise on Diet,” &c.


*Practical Observations on the Various Forms of Dyspepsia.* By Robert Dick, M. D., Author of a Treatise on the "Derangements, Primary and Reflex, of the Organs of Digestion," "A Treatise on Diet," &c.

"I am of opinion that we cannot bestow too much pains on the consideration of AFFECTIONS of the STOMACH, as we find, that, next to the Pyrexiæ, they are the most frequent occurrences in practice."—Cullen.

Having elsewhere treated, somewhat fully, the physiological questions connected with the subject of indigestion, my purpose, in the following papers, is to confine myself strictly to practical matters, and to handle these in the concisest manner possible. I shall begin with a very brief notice of the principal causes of indigestion.

The well-being of the stomach, in common with that of one or two other organs of the body, is not a little affected (though the remark may seem somewhat novel) by certain of our moral and intellectual, or perhaps I ought rather to say, our irrational tastes and habits. We never do our lungs conscious and voluntary injustice. Except from necessity, we never expose them to noxious influences or exertions. Such, however, is not the case with our treatment of our stomachs. Daily, perhaps every one of us, from custom or from want of self-restraint, gives his digestive organs more to do than our wants require: not uncommonly, from gustatory predilections more or less sophisticated, we select articles of diet and modes of cooking, less wholesome than others which might be chosen. In the causes now named a majority of digestive derangements undoubtedly originate.

Food and drink, improper in kind or in quantity, form the chief and most frequent causes of indigestion. Undue bulk is the first and simplest of the improper qualities of diet. Its operation is to a considerable extent mechanical, and its effect is to overcome the natural resiliency of the muscular coat of the stomach. This organ, as well as the gullet and intestines, is very contractile, insomuch that the sides of each are, when healthy, in a state of natural collapse. Thus, could we see food descending the œsophagus, we should observe that tube contracted above and below the morsel; could we inspect the stomach, we should see it closely embracing the food it was receiving, and only expanding in strict accommodation to its contents, between which and its walls no vacancy was permitted. Now, though a considerable capacity of distension is natural to the stomach, yet this may be exceeded by meals constantly large: and then the result after a time will be that the contractility of the muscular coat will be gradually overcome in a greater or less degree, so that the stomach though empty will not resume its natural calibre, but remain more or less flaccid.

The natural hue of the internal surface of the stomach is pale pink; its appearance soft and velvet-like. When the organ is undistended by food, the mucous coat, from being more ample than the others, falls into considerable plicæ. The interior of the organ is constantly lubricated with mucus, a semi-opaque and sometimes slightly saltish fluid. The gastric juice is distinct from mucus. The former never flows except at the stimulus of food. The mucus is neutral; the gastric juice acid. The latter, differently from the former, is limpid. Rising through the mucous surface it appears in shining specks, which bursting from the minute organs which secrete them, diffuse themselves over the whole surface of the stomach. As no sooner than food is swallowed, the alimentary mass is by a rallying movement of the stomach hurried from the fundus along the great curvature and back to the fundus along the lesser curvature; hence, the gastric juice, which flows all the while, is by this incessant tumbling of the contents of the stomach completely mixed up with the food.

From the description now given it will be easily understood how excessive meals operate injuriously. Over-distension of the stomach frequently repeated overcomes (as we have stated) the resiliency of the muscular coat. A species of muscular impotence ensues; the food lies comparatively motionless in the flaccid organ, and the important process by which the gastric juice is mixed intimately with the food, and brought in contact with the surfaces of the minutest fragments, is very imperfectly effected. Hence, just in proportion to the degree and permanence of over-distension of the stomach, is digestion tardy and imperfect, and as food, not transmitted downward, in due time acts, in consequence of chemical changes, and accumulations, as a morbid stimulant, various untoward consequences arise.

Over-distension and its ill effects may not be confined to the stomach, but excessive meals may extend their disastrous consequences over the whole alimentary canal. An alimentary mass larger than the system requires, and than can be duly digested, distends, in turn, the duodenum and small intestines, (interfering, meantime, with the free excretion of the liver by the common duct,) and, in consequence of the flaccid condition apt to be induced in the parts now named, impactions of the duodenum, and scybalous accumulations in the small intestines frequently occur. Another hurtful consequence of large, frequent, and stimulant meals, is the production of permanent hyperæmia of the mucous coat of the stomach, than which state nothing interferes more with the function of a secreting surface.

On the other hand, symptoms occasionally present themselves in practice that leave little doubt as to their being caused by a *contracted* state of the stomach and intestines. These occur in dyspeptics, who, harassed by the morbid sensibility of the stomach and bowels to almost every sort of food, have acquired a habit of taking aliment in only the smallest quantities and most concentrated forms, and into an almost total disuse of fluids. In these circumstances the stomach and bowels shrink amazingly, and any sudden increase in the volume of food is sure to cause in the first instance considerable suffering.

Every one has read the observations and the experiments of Dr. Beaumont on the stomach of St. Martin. The serious effects there detailed as following dietetic excesses are of a very curious and grave interest. At page 249, of Dr. Combe's edition of Dr. Beaumont's Experiments, we read the following:—"July 28th, nine o'clock, A. M., stomach empty; not healthy; some erythema and aphthous patches on the mucous surface. St. Martin has been drinking ardent spirits pretty freely for eight or ten days past. August 1st, 8 o'clock, A. M. Examined stomach before eating any thing; inner membrane morbid, considerable erythema, and some aphthous patches, on the exposed surface; secretions vitiated. Extracted about half an ounce of gastric juice, not pure and clear as in health; quite viscid. On the following day extracted one ounce of gastric fluids, consisting of unusual proportions of vitiated mucus, saliva, and some bile, tinged slightly with blood appearing to exude from the surface of the erythema, and aphthous patches which were tender and more irritrble than usual." On other occasions the secretions from the mucous membrane seemed to be entirely suppressed; sometimes they became so acrid as to smart and excoriate the edges of the aperture of St. Martin's stomach. Occasionally, during the use of improper aliment, the mucous membrane seemed abraded in parts, presenting the appearance of a blistered surface, with shreds of epidermis upon it. All these facts are alarmingly conclusive as to the effect of stimuli, imprudently and excessively used, in vitiating the secretions of the stomach, and otherwise disqualifying that organ for the due performance of its functions.

In St. Martin's case, the morbid causes above alluded to seemed sometimes to have *suspended* the secrelions of the stomach. Thus Dr. Beaumont not unfrequently makes mention of an irritating dryness of the internal surface of the organ, which characterized its deranged conditions, and during which the delicate papillæ were exposed, unprotected by the mucous secretion, to alimentary contact.

It is obvious that as improper food almost necessarily sooner or later ensures stomachic derangement, so stomachic derangement, by a similar necessity, involves duodenal disorder; the vitiated chyme of the stomach being to the duodenum what crude or irritating food is to the former. The connexion of the stomach with the duodenum may be stated as that of sympathy, but the relation of the duodenum to the stomach is something more. And the same remark applies to every inferior part of the digestive tube as regards parts superior to it. The duodenum has to contend not merely with its own deranged secretions, but with those also poured into it from the stomach, which last cause probably often primarily induces duodenal derangement, and materially aggravates it if already induced.

Functional disorder of the liver may long be the sole lesion in dyspepsia, but often the affection of the liver is something more than functional, and consists in a state of chronic inflammation. This term, the late Dr. Abercrombie very justly observes (page 365 of his Treatise on Diseases of the Stomach( is applied to a morbid condition of the liver which remains after an acute attack, *and a corresponding condition may come on gradually without any acute symptoms.*" Although the predetermined office of this organ is to secrete from venous blood, and although it is physiologically qualified for this function, yet it does not follow that the blood may not be so surcharged with imperfectly assimilated or other unusual ingredients, that even the liver, though expressly designed to rectify such states when existing in a *moderate* degree, may, in consequence of an *extremely* loaded and vitiated condition of the portal circulation, suffer such irritation as to lead to some chronic affection of the viscus. Cruveilhier, indeed, attributes hydatids of the liver to this cause; while M. Ribes supposes that a sub-inflamed state of the coats of the veins of the stomach and duodenum, caused by chronic derangements of these parts, and propagated by their veins to the liver, may be the source of certain sympathetic affections of this organ. Cruveilhier has seen inflammation of the rectum extend by the hemorrhoidal veins to the liver.

At page 393 of his work which we have just quoted from above, Dr. Abercrombie observes:—"It is probable that the bile may be increased in quantity, but it must, at the same time be admitted that our prevailing notions on the subject are rather hypothetical than founded on facts. I am not aware of any test by which we can judge with precision of its redundancy, and I must confess my suspicion that the term bilious stools is often applied in a very vague manner to evacuntions which merely consist of thin feculent matter mixed with mucus." And he quotes in support of this opinion from a paper of Mr. Tyler, in the Calcutta Transactions.

Without entering on the question whether and with what frequency, feculent are mistaken for bilious stools, and seeing that Dr. Abercrombie admits it to be probable that the bile may be increased in quantity, it would, I think, have been desirable that this eminent physician should have stated the grounds of his doubt as to the easy practicability of arriving at comparative certainty on this point.

In reference to this matter, we have Sir B. Brodie's, and Tiedemann and Gmelin's experiments. In the experiments of the two last, the excrements, after ligature of the biliary duct, were, I believe, in all cases white. We know, also, that many of the mechanical causes which produce jaundice, simultaneously induce clayish-colored stools. Dr. A. observes, in the same page from which the preceding extracts are made, that the bile, when mixed with the usual contents of the intestinal canal, imparts to them a bright yellow. It will give, doubtless, a corresponding tinge to the fæces; and the usual depth of this tinge, in healthy evacuations; being determined by an infinity of observations, it follows that if a very deep yellow, or a dark brown hue, be presented in the fæces, not to be accounted for by any thing remarkable in the recent ingesta, medicinal or dietetic; if, in addition, we find a more active, peristaltic movement of the intestines (another usual proof of the abundance of bile, as torpor of these is of its deficiency) giving rise to diarrhœa; we surely have sufficient evidence on which to ground scientific treatment, that the liver is secreting with unusual profuseness.

Dr Abercrombie, at page 385 of the work just quoted from, reprehends "the prevailing doctrine, or rather the prevailing phraseology, by which numerous symptoms are ascribed to disease of the liver on very vague and inadequate grounds;" and he regrets "the prevalence of this doctrine, and the indiscriminate use of mercury which has arisen from it." This jealousy of mercury Dr. William Thomson, formerly of Edinburgh, now of Glasgow, largely shares, as we learn from his work on the liver. I would merely here observe, that of all the abdominal organs the liver is the most frequently, easily, seriously deranged; the one which most readily takes on the successive phases of functional, chronic, structural disease. While this certainly is no excuse for the careless or rash practitioner, who fancies he sees hepatic disease in every stomachic or intestinal affection, yet Dr. Abercrombie's language may be apt to divert the attention of younger practitioners from an organ the chronic derangements of which he himself, in our opinion, much too qualifiedly, describes as being, "in a large proportion of them, beyond the reach of any human means."

As to the use of mercury, in disorders of the liver, we would here merely remark, that in many of these mercury is unquestionably the most efficient agent; and that objections founded on its abuse have no weight against its judicious use.

*The pancreas.*—Though we are, in a great measure, ignorant of the precise uses of the pancreas, yet there can be little doubt that it performs a part in the process of digestion. Dr. Baillie mentions a case of abscess of this organ, in which the symptoms before death consisted in wandering abdominal pains, in spasmodic affections of the abdominal muscles, in squeamishness, in stomachic distension. Disease of the pancreas may simulate, or rather be mistaken for, affections of the stomach, or of the left lobe and convex surface of the liver. It frequently causes epigastric pain and sickness, and sometimes jaundice, when structural disease, extending along its duct, involves the ductus choledochus, narrowing or obstructing that common conduit. Its enlarged bulk may also be detected through the stomach. and be mistaken for structural disease of that viscus; or else pressing on the ductus communis, or the duodenum, may cause obstruction of the former and impaction of the latter.

*Spleen.*—Of the function of the spleen we are even more ignorant than of that of the pancreas. But, doubtless, disorder of that function, in one way or another, affects digestion. Its proximity to the stomach renders it almost impossible that the spleen should be seriously disordered, and the stomach remain uninfluenced. Indeed, it is often through the stomach alone that affections of the spleen manifest themselves. Accordingly, we have cases in which obscure and generally mild dyspeptic symptoms alone announced the splenic disease which caused them, and which post mortem inspection unveiled. This organ is one of the most insensible organs of the body, so that the gravest and most extensive disorganization may go on in it without a sensation of pain being experienced. Even pressure on an inflamed spleen does not develope pain. Hepatic obstructions, by interrupting the portal circulation, may cause venous tumefaction of the spleen, in common with that of the stomach and pancreas. To this cause are probably owing those alternations of fulness and pain between the liver and this organ which Dr. Philip notices.

I have stated that errors either in the quantity or the quality of diet are by far the most frequent and efficient causes of dyspeptic derangements; yet it is scarcely necessary to observe that indigestion may owe its origin to almost any of the causes which produce disease in other organs of the body. There may, undoubtedly, be an original or acquired disposition to derangement of one or more of the principal organs of digestion, as there so frequently is to pulmonary, renal, or other disease. Most persons have what may be called one weak point in their system, one organ, or set of organs, more apt than others to fall into irregularity of action: accordingly, with some persons, the stomach, the liver, or the colon, or all these organs, costs them trouble and attention; with others, cold excites irritation, not in the pulmony, but the gastric mucous membrane; others on exposure to damp and raw air, fail not to suffer from catarrh, complicated with marked biliary derangement; others, on any occasion of unusual thought or anxiety, are sure to have what, in familiar but sufficiently intelligible language, is called a fit of indigestion. Hence, then, the disease in question may arise from changes of temperature, from fatigue, from mental labor, from moral excitement, besides a thousand other causes capable, or not capable, of being appreciated.—*Bulletin Med. Sci. for July.*

